# Efficacy and Safety of Topical Timolol Eye Drops in the Treatment of Myopic Regression after Laser In Situ Keratomileusis: A Systematic Review and Meta-Analysis

**DOI:** 10.1155/2015/985071

**Published:** 2015-12-21

**Authors:** Xiaochen Wang, Guiqiu Zhao, Jing Lin, Nan Jiang, Qian Wang, Qiang Xu

**Affiliations:** Department of Ophthalmology, The Affiliated Hospital of Qingdao University, Qingdao 266000, China

## Abstract

*Aims*. The aim of this study was to assess the efficacy and safety of timolol in the treatment of myopic regression after laser in situ keratomileusis (LASIK).* Methods*. We searched MEDLINE, CENTRAL, EMBASE, China National Knowledge Infrastructure (CNKI), and Chinese Biological Medicine (CBM) from the inception to July 2015 for relevant randomized controlled trials that examined timolol therapy for myopic regression. The methodological quality of the studies included was assessed using the Revman 5.3 software.* Results*. We included six clinical trials involving 483 eyes in this review, including 246 eyes in treated group and 237 eyes in controlled group. We observed statistically significant improvements on the postoperative SE in the 3 months. However, the change of CCT was not statistically different between the control group and the experimental group. There were fewer cases of IOP, UDVA, and CDVA in treated group having significant difference from the controlled group.* Conclusions*. Topical timolol could be an effective treatment for reduction of myopic regression especially the spherical errors after myopic LASIK. Further RCTs with larger sample sizes for these trials are warranted to determine the efficacy and limitation for myopic regression after LASIK.

## 1. Introduction

Laser in situ keratomileusis (LASIK) is thought to be an effective and safe refractive surgical procedure for the high myopia [1]. Along with the continuous renewal of equipment instrument and the continuous improvement of surgical technique, most postoperative patients obtained satisfactory results. However, at least 28% of refractive surgery patients still experience myopic regression [2–5].

In previous studies, “regression” was defined as a 0.25-diopter (D) or greater myopic shift occurring between follow-up visits [4–7]. Nevertheless, the mechanism for refractive regression is very complicated and is not fully understood. The main possible explanations for regression are focused on the forward shift of the cornea [8–11]. It has been suggested that intraocular pressure- (IOP-) lowering agents or the corneal biomechanical change can decrease and alleviate myopic nonselective B-blocker with carbomer and polyvinyl alcohol [12]. Timolol provides ocular comfort and lubrication and also increases retinal and optic nerve perfusion. It can reduce IOP by decreasing aqueous humor production and has no obvious side effects. Because of the properties noted above, topic timolol eye drops are indicated for the treatment of myopic regression. A number of clinical trials had been conducted to evaluate timolol's effectiveness and safety. However, the results were inconsistent; therefore, we set out to conduct a systematic review and meta-analysis to assess the evidence for treating regression.

## 2. Materials and Methods

### 2.1. Search Strategy and Selection Criteria

We performed our research with MEDLINE, CENTRAL, EMBASE, CBM, and CNKI for randomized controlled trials (RCTs). The search terms used were “Timolol AND (myopic OR regression OR regressive)”. Furthermore, we reviewed citations in the retrieved articles to search for additional relevant studies.

*Inclusion and Exclusion Criteria*. RCTs were eligible for inclusion if the following criteria were satisfied:

1. There are controlled clinical trials, including retrospective studies and prospective studies such as randomized controlled trials (RCTs).
2. There is confirmed diagnosis of high myopic, spherical equivalent (SE) ≥ −6.00 D; age of patients is 19 years or more.
3. Studies that reported the follow-up results beyond 2 weeks concerning LASIK treatment for myopia are included.
4. Patients were subjected to topical timolol eye drops daily for more than two weeks.
5. Treatment with topical timolol eye drops was compared with artificial tears, placebo (vehicle), with no topical treatment.
6. We included any RCTs that examine at least one of the following outcomes: IOP, spherical equivalent, CCT, UDVA, and CDVA.

Studies were excluded based on the following criteria:

1. Patients had a history of other ocular diseases, especially the glaucoma, active inflammation.
2. Outcomes or data are presented in a format that cannot be extracted for analysis.
3. Patients had the refractive surgery but not the LASIK.

### 2.2. Data Extraction and Assessment of Bias Risks

All articles were read by two independent reviewers (Xiaochen Wang and Qian Wang) independently who implemented the data extraction according to the inclusion criteria. We use a standardized form to record data on the authors of the study, year of publication, country of origin, sample size, gender, mean age, duration of follow-up, and outcome measures. The risks of bias in the included studies were assessed according to the recommended methods of the Cochrane handbook. We evaluated random sequence generation and allocation concealment (selection), masking of participants and personnel (performance bias), masking of outcome assessment (detection bias), and incomplete outcome data (attrition bias). Two authors (Xiaochen Wang and Qian Wang) independently assessed the risks of bias and any disagreements were resolved by discussion to reach a consensus among the investigators.

### 2.3. Statistical Analysis

We used the Review Manager 5.3 to perform our meta-analysis. We calculated the weighted mean difference for continuous data. We used the SMD to analyze the results on a uniform scale. The absolute value is interpreted together with the *P* value and confidence intervals (CI). We evaluated the statistical heterogeneity by Cochrane *χ* ^2^ tests and qualified it by calculating the *I* ^2^ statistic. If there was any significant heterogeneity between studies (*I* ^2^ > 50%), a random effects model was used to pool the data; otherwise a fixed effect model was used. We considered conducting a sensitivity analysis by excluding studies which were at high risk of bias in the protocol, but the current study does not include many more meta-analyses so the sensitivity analysis was not done. If possible we will do further sensitivity analysis, so that we can judge the importance of review results to crucial decisions and assumptions that we have made during the review. In addition, we performed subgroup analysis to identify the differences in different follow-up.

## 3. Results

**Figure 1:**
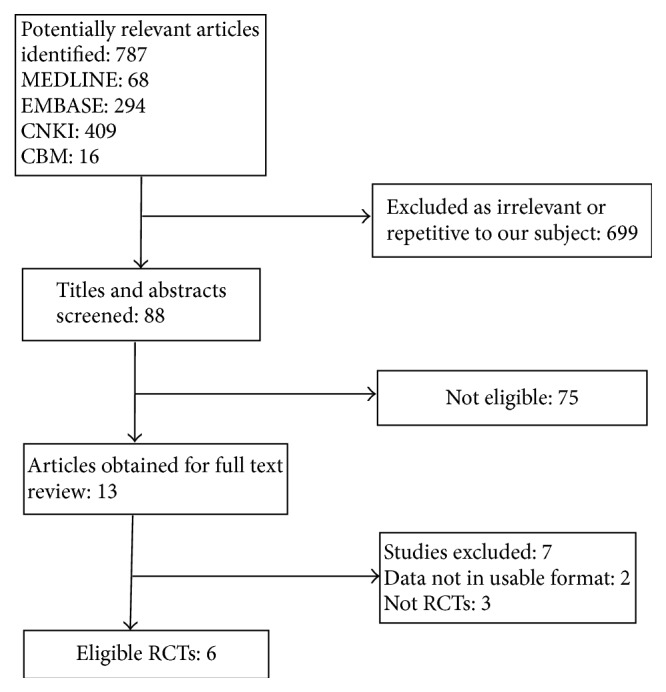
Flowchart of the trial selection process. RCT: randomized clinical trial.

We identified a total of 787 titles and abstracts from the literature, and we retrieved 13 full texts for review. We finally included 6 RCTs in our systematic review and meta-analysis [13–18] (Figure 1).

### 3.1. Characteristics of Included Studies

A total of 398 patients with LASIK were enrolled in these studies.  Table 1 summarizes the main demographic characteristics of the included trials.  Table 2 shows the clinical profiles of the eligible studies. The 6 included studies included 4 prospective studies [13, 15, 16, 18], involving a total of 471 eyes, including 240 in timolol group and 231 eyes in control group. The six articles were published in the last five years. The mean age of participants was 24.24 years, and 49.5% were male. The follow-up period ranged from 7 days to 12 months. Four studies [13–16] use 0.5% timolol. Topical timolol was prescribed twice daily in five studies except only one study [18]. The mean pre-LASIK SE is −7.575 D. There is no difference between the two groups.

### 3.2. Risks of Bias in Included Studies

**Figure 2:**
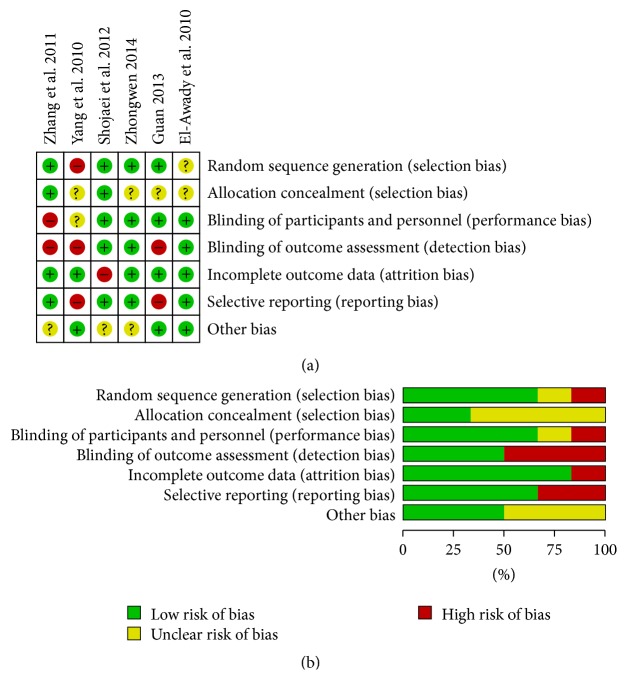
(a) Risk of bias summary: authors' judgments about each risk of bias item for each included risk. (b) Risk of bias graph: authors' judgments about each risk of bias item presented as percentages across all included studies.

Figures 2(a) and 2(b) summarize the risks of bias assessment of the 6 included studies. The adequate methods of sequence generation were used to minimize selection bias in 4 of the studies [13–16]. For performance and detection biases, 4 studies [13–15, 18] reported using blinding method to performance and outcome assessment. For attrition bias, only 1 trial [15] had high loss to follow-up and was judged from high risk of bias. In the other studies, attrition bias was considered to be possible. In the included trials, reporting bias was not considered to be a major problem but it was always difficult to evaluate it sufficiently.

### 3.3. Outcome Measures

#### 3.3.1. Spherical Equivalent

**Figure 3:**
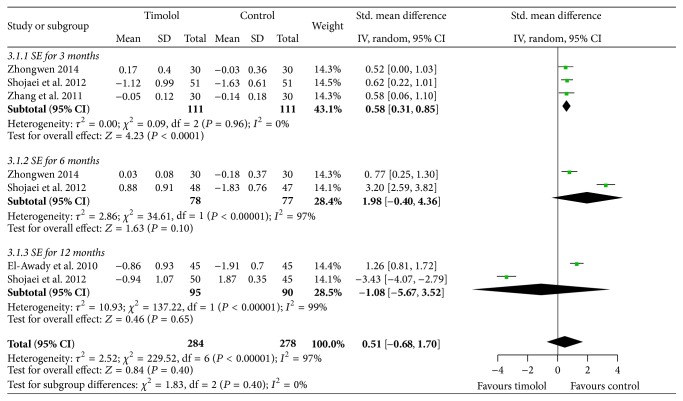
Forest plot comparing the spherical equivalent refraction in timolol and control groups. SD: standard deviation; IV: inverse variance; CI: confidence interval.

Four studies reported the final refractive spherical equivalent after being treated for 3 months, 6 months, and 12 months, respectively, and used the random effects model to analyze the data for heterogeneity (*I* ^2^ = 0%, 97%, 99%). There was statistically significant difference between the two groups in the follow-up for 3 months (SMD = 0.58, 95% CI = 0.31 to 0.85; *P* < 0.0001). However, in 6 months (SMD = 1.98, 95%  CI = −0.40 to 4.36; *P* = 0.1) and 12 months (SMD = −1.08, 95%  CI = −5.67 to 3.52; *P* = 0.65), there were no differences between the two groups (Figure 3).

#### 3.3.2. Central Corneal Thickness

**Figure 4:**
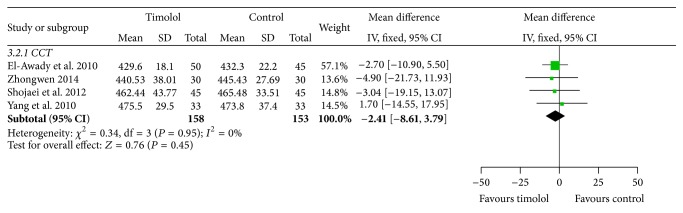
Comparison of central corneal thickness in patients with myopic regression after LASIK.

The data of the central corneal thickness were used the fixed effects model to analyze the heterogeneity (*I* ^2^ = 0%). The change of CCT was not statistically different between the two groups (MD = −2.41, 95%  CI = −8.61 to 3.79; *P* = 0.45) (Figure 4).

#### 3.3.3. Intraocular Pressure

**Figure 5:**
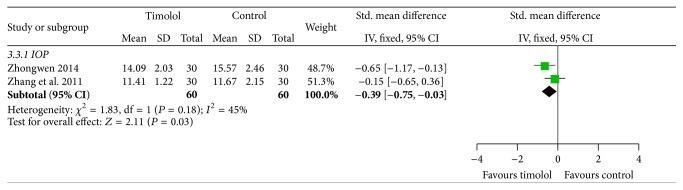
Intraocular pressure in timolol and controls groups.

There were 2 studies [13, 16] that reported the intraocular pressure, showing significant difference between the two groups (SMD = −0.39, 95% CI = −0.75 to −0.03; *I* ^2^ = 45%; *P* = 0.03) (Figure 5).

#### 3.3.4. UDVA

**Figure 6:**
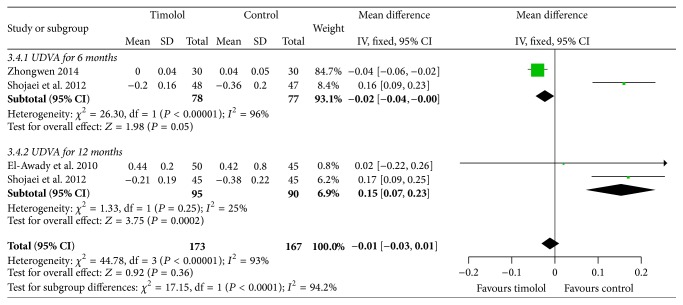
Comparison of logMAR UDVA between the two groups in different time.

Each of the 2 studies reported the logMAR UDVA that used the fixed effects model to analyze the data for heterogeneity (*I* ^2^ = 96%, 25%) in different time points. There were significant differences between the two groups in the follow-up for 6 months (MD = −0.02; 95%  CI = −0.04 to 0.00; *P* = 0.05) and 12 months (MD = 0.15; 95% CI = 0.07 to 0.23; *P* = 0.0002) (Figure 6).

#### 3.3.5. CDVA

**Figure 7:**
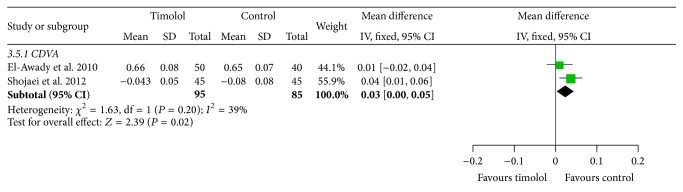
Comparison of logMAR CDVA between the two groups in two studies.

There were 2 studies [15, 18] that use the logMAR CDVA to measure the outcome and then we used the fixed effects model to analyze the data for heterogeneity (*I* ^2^ = 39%); the results show that it is significantly different between the two groups in the follow-up for 12 months (MD = 0.03; 95% CI = 0 to 0.05; *P* = 0.20) (Figure 7).

### 3.4. Heterogeneity and Publication Bias

Some outcomes displayed great heterogeneity. The heterogeneities of SE and IOP were significant, and dropping eligible studies by hand and metaregression have not provided good results. Maybe it is because of the different measure tools. No significant publication bias was demonstrated in the funnel plot.

## 4. Discussion

Meta-analysis attempts to analyze and combine the results of previous reports [19]. This systematic review provided a critical overview of previous clinical reports and combined effect measures of timolol in multiple small clinical trials to increase statistical power. It included six trials using timolol to prevent and treat the myopic regression after LASIK. All trials were implemented in developing countries because of the higher incidence than developed countries. There are still no large multicenter randomized trials to assess the efficacy and safety of timolol on the treatment of myopic regression.

As a common clinical phenomenon, refractive regression can affect the predictability, efficiency, and long-term stability of refractive surgery and lead to deterioration in visual performance and even seriously affect the surgical curative effect and patients' satisfaction. So the prevention and treatment of refractive back after the surgery are very important to the quality of patient's life in the future. Nevertheless, there are no unified and effective methods in the treatment of myopic regression. Secondary surgery is an inacceptable method for patients and doctors; both of them have very big challenge. In contrast, effective drug treatment is a lower risk more easily accepted by patients.

There have been many factors which associated with myopic regression after LASIK, including preoperative refraction [4, 5, 19–23], preoperative keratometry [20, 21, 24], corneal thickness [11, 23], flap thickness [24, 25], ablation depth [21], optical zone size [21, 26], chronic dry eye [27], age [21], surgeon, IOP [20, 22], postoperative undercorrection, and humidity. The occurrence of refractive regression has the relation with the corneal wound healing response, the destruction of the corneal biomechanics structural integrity, and relatively high intraocular pressure and closely related to the occurrence of postoperative dry eye. There is a debate according to the role of CCT in myopic regression. Kamiya and associates [28] present a theory to assess the effects of nipradilol, an IOP-lowering agent; Pan et al. [11] compared regressive eyes with nonregression after LASIK and indicated that refractive regression after LASIK might be mainly induced by corneal protrusion, rather than central corneal thickening. That is what happens with any refractive procedure or flap; the corneal biomechanics changing may be a factor of the myopic regression. From these studies, we conclude that LASIK can lead to the destruction of the corneal biomechanics structural integrity, corneal injury repair reshaping in the early postoperative stage, the strength of the corneal resistance reduced, intraocular pressure remaining unchanged, and intraocular pressure greater than the corneal resistance. Therefore, the bulging forward of the cornea that caused corneal diopter increasing is the primary cause of myopia refractive regression after LASIK [29].

Timolol as a kind of commonly used ocular hypotensive agent has a good clinical effect. So far, however, because of LASIK postoperative corneal shape to the process and the fact that its mechanism is not clear, when we use timolol postoperatively, the use of the drug dose and time have not yet been determined. So this meta-analysis for the effects of timolol for prevention and treatment of refractive regression made a systematic review.

The results of this meta-analysis show that we can use the timolol eye drops to prevent and treat myopic patients undergoing LASIK and occurring refractive regression. The SE in 5 trials mentioned have statistical differences between the timolol groups and the controlled groups (*P* < 0.05); it declared the fact that the IOP after LASIK is one of the reasons for the SE decline. These results indicate that IOP reduction may have induced a backward shift of the cornea and reduction of corneal refractive power, resulting in refractive improvement in post-LASIK eyes. It may be that the morphologic properties of the cornea are affected easily by subtle changes in IOP and atmospheric pressure when corneal rigidity is impaired by flap manipulation and laser ablation such as LASIK. But the CCT in four trials have no significant differences between the timolol groups and the controlled groups (*P* > 0.05). The result indicated that the corneal hydration may not play an important role in the refractive changes in these studies. The IOP, UDVA, and CDVA in treated groups are significantly different from those in the controlled groups (*P* < 0.05). Shojaei et al. [15] concluded that the SE, UDVA, and CDVA improved in patients with myopic regression after timolol application compared with the control group and improvement lasted for at last 6 months after timolol was stopped. Zhongwen [13] also chooses the follow-up for 6 months after LASIK to compare because myopic regression can be stable in 6 months. The timolol dose is 0.5% gel that can be better for patients.

This meta-analysis still has some limitations. First, the studies only have six trials; it is not enough to analyse the outcome and it is easy to produce bias. In addition, some parameters had relatively large heterogeneity. The heterogeneities of SE and IOP were not explained due to different surgical techniques, different methods of measurement, or different follow-up periods in different trials. However, we still believe that the results of this meta-analysis are useful, because the meta-analysis includes a relative large number of studies and cases which provide a strong power and the consonance of previous results and sensitivity analysis.

In conclusion, timolol was effective for reduction and improvement of myopic regression especially the spherical errors after myopic LASIK. Importantly, further RCTs with large sample size are needed and the search for more effective and cheaper interventions for this trial would be necessary.

## Figures and Tables

**Table 1:** Demographic characteristics of eligible studies.

Study (year)	Country	Population	Gender (male : female)		Mean age (Yr) ± SD	
			Timolol	Control	Timolol	Control
Zhongwen 2014 [13]	China	60	NS	NS	24.47 ± 5.45	25.07 ± 6.23
Guan 2013 [14]	China	60	18 : 12	16 : 14	20.0 ± 7.50	22.0 ± 5.50
Shojaei et al. 2012 [15]	Iran	90	9 : 36	15 : 30	33.31 ± 10.90	34.42 ± 8.57
Zhang et al. 2011 [16]	China	60	NS	NS	25.37 ± 6.13	24.53 ± 2.31
Yang et al. 2010 [17]	China	53	NS	NS	NS	NS
EI-Awady et al. 2010 [18]	Egypt	75	NS	NS	NS	NS

SD: standard deviation; Yr: years; NS: data not available.

**Table 2:** Clinical characteristics of eligible studies.

Study (year)	Study design	Conc. of timolol (%)	Timolol regimen and duration	Follow-up	* *Mean pre-LASIK SE ± SD, diopters		* *Mean pre-LASIK IOP, mmHg	
					Timolol	Control	Timolol	Control
Zhongwen 2014 [13]	Prospective	0.5	Twice a day for 1 mo	1 wk/1 mo/ 3 mo/6 mo	−7.00 ± 0.77	−7.32 ± 1.10	16.33 ± 2.69	16.90 ± 3.00
Guan 2013 [14]	NS	0.5	Twice a day for 3 mo	3 mo	−5.85 ± 2.52	−5.64 ± 2.31	14.65 ± 2.35	15.45±2.13
Shojaei et al. 2012 [15]	Prospective	0.5	Twice a day for 6 mo	3 mo/6 mo/ 12 mo	−8.10 ± 3.41	−4.87 ± 1.88	12.73 ± 1.43	12.38 ± 1.65
Zhang et al. 2011 [16]	Prospective	0.5	Twice a day for 1 mo	1 wk/1 mo/ 3 mo	−4.94 ± 1.09	24.53 ± 2.31	15.22 ± 1.78	15.11 ± 2.53
Yang et al. 2010 [17]	NS	0.025	Twice a day for 2 wk	2 wk	−7.01 ± 3.04	−6.53 ± 2.40	NS	NS
EI-Awady et al. 2010 [18]	Prospective	0.1	Once a day for 12 mo	12 mo	NS	NS	NS	NS

SD: standard deviation; Yr: years; mo: months; wk: weeks; Conc.: concentration; NS: data not available.
